# Verbal Emotional Disclosure of Traumatic Experiences in Adolescents: The Role of Social Risk Factors

**DOI:** 10.3389/fpsyg.2017.00372

**Published:** 2017-03-14

**Authors:** Silvia Pérez, Wenceslao Peñate, Juan M. Bethencourt, Ascensión Fumero

**Affiliations:** Psicología Clínica, Psicobiología y Metodología, Universidad de La LagunaLa Laguna, Spain

**Keywords:** verbal emotional disclosure, traumatic experiences, psychopathological symptoms, adolescents at risk, social exclusion

## Abstract

It is well-known that traumatic events and adverse life situations are very important in both physical and psychological health. Prevalence studies suggested that adolescents experience at least one potentially traumatic event before reaching age 18. The paradigm of research centered on expressive writing has evidenced the beneficial effects that the emotional disclosure of previous traumas produces on physical health and psychological adjustment. The aims of the study are threefold: determining the prevalence of adverse or traumatic events; examining the extent to which psychopathological symptoms developed in those exposed to traumatic events; and exploring an verbal emotional disclosure (VED) paradigm in which variations on time spent talking about traumatic experiences to others resulted in a reduction of the psychological impact of trauma in a sample of Spanish adolescents. 422 volunteer adolescents participated, 226 boys and 192 girls, from 10 to 19 years old, all of them living in Tenerife. The mean age was 14.8 years (*SD* = 1.83). All of them completed the instruments used to assess the psychological impact of traumatic experiences and VED. The main results indicated that 77% of the participants had suffered a traumatic situation. The participants who have been exposed to traumatic events scored significantly higher in measures of post-traumatic stress, disorder, intrusive thoughts, avoidance behaviors, anxiety and depression, compared to those without trauma. Furthermore, results show a decrease in symptomatology scores as a function of time spent disclosing emotional experiences to others, particularly when disclosure occurred several times. In conclusion, stressful events or traumatic experiences and their concomitant emotional effects are highly prevalent in adolescents, and repeated VED to others appears to ameliorate their impact. VED shows greater therapeutic benefits when adolescents narrate the experience on several occasions and in an extensive way.

## Introduction

The experiencing of traumatic, painful, or stressing situations in a person’s life is not harmless, and may cause serious difficulties in psychological adjustment (Walter and Bates, 2012). When these experiences become part of everyday life, the probability of suffering a psychological disorder is very high (Campo-Arias et al., 2014). The impact of the traumatic situations or adverse life events during childhood and adolescence may be much more significant. Adolescence is a potentially significant period in developing stress and coping processes (Hollenstein and Lougheed, 2013). It is well-documented that exposure to multiple traumatic events, as well as other risk behavior, such as gambling or substance use, are common among juvenile offenders (Lee et al., 2012). The importance of the adverse events in adolescence may be due to several reasons. First, change is viewed as an inherent component of stress, and adolescence is characterized by changes in biological functioning, cognitive development, social roles, and social environments. Second, because adolescence is a period of transition, change and adaptation. Third, cognitive and social development during adolescence may make it an optimal time for learning new coping skills to reduce the adverse effects of stressful events (Compas et al., 1985).

Adolescents at risk of social exclusion constitute a population especially sensitive to traumatic experiences. They lacked the necessary moral or material assistance which must be provided by those who are supposed to take care of them; they suffered abandonment, physical and/or psychological abuse, regular alcoholism or drug addiction in members of the family, sexual abuse, serious violent behavior or attitudes by relatives or third parties in the family unit, incitement to mendicancy, delinquency, prostitution, or to any other form of economic or sexual exploitation.

Studies about prevalence of exposure to trauma suggest that many children and adolescents experience at least a potentially traumatic event before reaching 18 years of age (Alisic et al., 2014). It is estimated that approximately 1 in 4 youths will experience some type of substantive trauma, such as physical abuse, sexual abuse or domestic violence during his or her developmental years (Duke et al., 2010). Adolescents are considered an at-risk population due to the serious negative consequences associated with exposure to trauma, from PTSD (post-traumatic stress disorder) to other anxiety disorders, behavior issues, depression, substance abuse, and risk behavior for health (Shi et al., 2016; Zhen et al., 2016). It has been demonstrated that a greater number of adverse events during childhood result in worse health in adults (Barboza Solis et al., 2015). With four or more adverse events in childhood, the risk of suffering several medical conditions increased exponentially (Perry, 2014).

The importance of language in the processing of trauma and subsequent health outcomes has been recognized. There is empirical support for a relationship between emotional disclosure about traumatic events and health outcomes (Pennebaker et al., 1988; Pennebaker, 1989). Specifically, verbal disclosure in comparison with writing achieved the greatest improvements in cognitive change, self-esteem, and adaptive coping strategies (Esterling et al., 1994). Also, most forms of psychotherapy include trauma disclosure. The reason should be in using language to label an emotion, and an experience creates a structure, which facilitates the assimilation and understanding of the event, and thus the reduction of emotional arousal that is detrimental to physical and psychological health (Pennebaker et al., 1997).

There is evidence of significant benefits in emotional health, physical health, positive changes in the immune system, and in the general psychological functioning after emotional self-disclosure or communication to others (Frattaroli, 2006; Lumley et al., 2014). However, results are less consistent for participants with psychological difficulties (Baikie et al., 2012; Travagin et al., 2015). While some studies have supported written emotional disclosure in students with a history of trauma (Ironson et al., 2013), benefits appear to be limited in samples of participants with a negative image of their own body (O’Connor et al., 2011; Lafont and Oberle, 2014), adults who suffered child abuse, exhibit symptoms of depression and post-traumatic stress, or had lost a relative (Baikie et al., 2012; Meston et al., 2013; Unterhitzenberger and Rosner, 2014). There have been significant effects on positive affect, negative affect, and level of depression, after an intervention based on expressive writing. However, anxiety, intrusive thoughts, and avoidance behaviors did not change (Del Pino et al., 2016).

In spite of the promising results with the adult population, there is scant evidence about the effectiveness of emotional disclosure in the adolescents. Findings indicate the beneficial effects of the use of emotional disclosure in children and adolescents on the symptoms of internalizing and externalizing behaviors (Zajac et al., 2015), a significant increase in optimism, a decrease in negative affect, the development of better coping strategies, such as positive reframing, and optimistic thinking (Margola et al., 2010; Graham-Bermann et al., 2011), and the strengthening of the academic self-concept (Facchin et al., 2014). Travagin et al. (2015) carried out a meta-analysis that evaluated the effects of expressive writing on adolescents; results suggest that emotional disclosure may help reduce internalization problems, behavior issues, somatic complaints, as well as improve social adjustment, school participation and performance. Briefly, emotional disclosure tends to produce significant improvements in the well-being of adolescents.

The effect of emotional disclosure on adolescent aggressive behavior and emotional lability has also been shown (Kliewer et al., 2011). Results suggest that emotional disclosure was an effective way of helping students deal with the stress factors they experienced, with those adolescents most exposed to violence being the most benefited. Furthermore, the adolescents who reported body dissatisfaction, and disclosed their emotional experiences through expressive writing showed a reduction in thin ideal internalization, personal dissatisfaction, and psychosocial deterioration (Graham-Bermann et al., 2011). Besides, adolescents who regarded problems with classmates as a stress factor improved their personal well-being and social adjustment in the long term, after emotional disclosure (Travagin et al., 2015).

This study is aimed at establishing to what an extent a population of adolescents is at high risk of experiencing traumatic situations, and whether psychopathological symptoms, such as PTSD, intrusive thoughts, avoidance behaviors, anxiety, and depression developed in a sample of adolescents exposed to traumatic events. The study also explores the extent to which variations on time spent talking about traumatic experiences to others modulates the psychological impact of trauma.

## Materials and Methods

### Participants

The sample consisted of 422 volunteer adolescents who were recruited from different Tenerife schools; 101 of them were under the Child Protection System, declared by the Dirección General de Dependencia, Infancia y Familia (DGDIF) (Dependency, Childhood and Family Department) of the Government of the Canary Islands, to be unquestionably abandoned, and admitted to Protection Centers, and 321 were enrolled at Secondary Education Institutes. 228 (54%) males, and 194 (46%) females, aged between 10 and 19 years. The mean age of the total sample was 14.8 years (*SD* = 1.83). The sample includes volunteers with various levels of education, from 4th grade Primary School to 2nd year High School or Intermediate Professional Education, the most common being 2nd year Compulsory Middle School. The study was approved by the Research and Ethics Committee at Universidad de La Laguna.

### Procedure

The collection of information started with the recruitment of the sample, which was carried out through the following procedure: in the first place, for the participation of adolescents under the Child Protection System, authorization was sought from the Unidad Orgánica de Infancia y Familia (Organic Childhood and Family Unit), a competent body in Foster Care, of the Island Council of Tenerife. After the authorization to conduct the study was granted, every participant voluntarily agreed to participate, and the collection of information started at the Protection System centers. Secondly, for the collection of data from adolescents enrolled at secondary education institutes, appointments were arranged with educational centers in the island. Following submission of the study to the directors in charge of each educational center, all the participants individually completed every assessment instrument in their classrooms. Different groups were used. The first one included those participants who reported any traumatic situation; the purpose was to detect the existence of symptoms compared to those who had not been exposed to this kind of situations. From the second sample, information was collected about whether they had narrated the experience, and for how long; the purpose was to know whether the psychological impact of the stressing event was lesser in participants who disclosed their emotional experiences.

### Measures

The socio-demographic questionnaire records the age, sex, and educational level of the study participants.

The verbal emotional disclosure (VED) scale is a self-report as a semi-structured interview, developed to determine the experience of stressing, painful and/or traumatic situations, composed of questions in which adolescents reported (i) their traumatic experience; (ii) whether they spoke with somebody about it; (iii) who those persons were (teachers, parents, psychologists, etcetera); and the duration of disclosure These questions were developed according to the recommendations of systemic reviews and meta-analyses (Frattaroli, 2006), which indicated an increase in the effectiveness of emotional disclosure when it occurs repeatedly, and spending more time talking about the adverse or traumatic event to others. In that sense, no psychometric data are available and every question was considered an autonomous variable.

The Child Post-Traumatic Stress Disorder Symptom Scale (CPSS; Foa et al., 2001) assesses the presence of post-traumatic stress symptoms in children from 8 to 18 years of age, with a known trauma history. The scale is based on the DSM-IV diagnostic criteria, and consists of 17 Likert-type items referring to how frequently symptoms of this disorder appear. This instrument can be used as a self-report, or as a structured clinical interview. It is composed of three subscales: re-experiencing (five items), avoidance (seven items), and arousal (five items). Meyer et al. (2015) reported that the the Spanish version of the total symptom scale demonstrated excellent internal consistency (α = 0.88), and moderate to good consistency within the sub-scale symptom categories (i.e., re-experiencing, avoidance, and hyper-arousal) (range: α = 0.71–0.84).

The Impact of Event Scale, Revised (IES-R; Weiss and Marmar, 1997) consists of 15 dichotomous items; seven evaluate answers relating to intrusion, and eight relating to avoidance, about the most stressing recent life event. The scale assesses two factors: intrusive thoughts as regards the experienced event, and the avoidance answers associated with the presence of those events. The Spanish version of this scale showed adequate psychometric adjustments; Cronbach’s alpha for the overall scale 0.94, intrusion 0.95, and avoidance 0.87; test–retest reliability coefficients = 0.35, 0.36, 0.28 for total scores, intrusive thoughts, and avoidance behaviors, respectively (Báguena et al., 2001).

The Hospital and Anxiety and Depression Scale (HADS; Zigmond and Snaith, 1983) consists of 14 items (seven for anxiety, and seven for depression) distributed in two subscales. It is centered on the emotional and cognitive aspects of these two disorders. The Spanish version of the scale has shown adequate psychometric adjustments; Cronbach’s alpha 0.74, 0.59, y 0.76 for anxiety, depression, and overall scale, respectively (Gil et al., 2015).

### Data Analysis

A descriptive design was made ex post facto. The predictor variables used were the presence or non-presence of traumas, whether VED occurred, and the time spent making the disclosure, and the criterion variables analyzed: PTSD, anxiety, depression, intrusive thoughts, and avoidance behaviors.

A descriptive analysis of traumatic experiences was made, which was followed by a contrast of means to examine the symptomatology associated with exposure to the traumatic event.

Multivariate analysis of variances (MANOVAs) were conducted to analyze the effect of the emotional trauma re-experiencing on the psychopathological variables, and to check whether differences appeared in the psychopathological variables according to the time the adolescents spent on VED, whether they talked about it for a moment, more than half an hour, or on several occasions.

## Results

**Table 1** shows the descriptive statistics of the variables analyzed in the study. The results about prevalence indicated that 77% of the participants (*n* = 324) had suffered a traumatic situation.

**Table 1:** Descriptive statistics of groups with and without trauma experience.

	With trauma experience (*n* = 324)		Without trauma experience (*n* = 98)	
	*M_1_*	*SD_1_*	*M_2_*	*SD_2_*
PTSD	25.11	13.98	16.01	12.28
Intrusion	8.34	5.69	5.14	5.48
Avoidance	9.58	5.60	6.18	5.91
Anxiety	7.40	4.30	5.02	3.60
Depression	5.81	3.81	5.81	3.55

*M_*1*_, group 1 mean; SD_*1*_, group 1 standard deviation; M_*2*_, group 2 mean; SD_*2*_, group 2 standard deviation; PTSD, post-traumatic stress disorder.*

The comparison between groups showed that the participants who have been exposed to traumatic events scored significantly higher in PTSD, intrusive thoughts, avoidance behaviors and anxiety, compared to those without exposure to traumatic events but not in depression (**Table 2**).

**Table 2:** Difference in symptomatology between groups with or without trauma experience.

	Without trauma experience (*n* = 98)		With trauma experience (*n* = 324)		*t(p)*
	*M_1_*	*SD_1_*	*M_2_*	*SD_2_*	
PTSD	14.21	13.19	25.49	15.15	-6.43^∗∗∗^
Intrusion	4.67	5.88	8.39	5.90	-5.06^∗∗∗^
Avoidance	5.60	6.40	9.68	5.93	-5.34^∗∗∗^
Anxiety	4.79	3.76	7.45	4.47	-5.04^∗∗∗^
Depression	5.80	3.83	5.81	3.98	-0.11 n.s.

*M_*1*_, group 1 mean; SD_*1*_, group 1 standard deviation; M_*2*_, group 2 mean; SD_*2*_, group 2 standard deviation; PTSD, post-traumatic stress disorder. ^∗∗∗^*p* < 0.001; n.s., non-significant.*

The MANOVA tests conducted with the adolescents’ information about their VED, showed statistically significant differences in depression between the group who disclosed their emotional experiences and the group who did not, that is, levels of depression decreased when VED had occurred [*F*(1,219) = 4.63, *p* = 0.03]. **Table 3** contains the descriptive statistics of PTSD symptoms, intrusive thoughts, avoidance behaviors, anxiety, and depression in both the group who disclosed their emotional experiences, and the group who did not.

**Table 3:** Descriptive statistics of groups based on whether verbal emotional disclosure has taken place or not.

	With VED (*n* = 233)		Without VED (*n* = 76)	
	*M_1_*	*SD_1_*	*M_2_*	*SD_2_*
PTSD	25.85	14.75	26.11	15.43
Intrusion	8.30	5.76	8.62	6.13
Avoidance	9.59	5.90	10.37	5.76
Anxiety	7.80	4.61	7.16	4.50
Depression	5.56	3.84	6.60	4.27

*M_*1*_, group 1 mean; SD_*1*_, group 1 standard deviation; M_*2*_, group 2 mean; SD_*2*_, group 2 standard deviation; PTSD, post-traumatic stress disorder; VED, verbal emotional disclosure.*

Of the total sample that reported to have suffered a traumatic experience (324), cases with missing values have been taken away, resulting in a sample made up of 309 (233 with VED and 76 without VED). A new MANOVA was conducted to check whether there were significant differences in the psychopathological variables as a function of time participants spent disclosing their stressful or traumatic events to others, that is, whether they had only made a comment, had spent more than half an hour, or had done so several times. **Table 4** shows the means and the standard deviations of measures of PTSD symptoms, intrusive thoughts, avoidance behaviors, anxiety and depression. Results show a decrease in symptomatology scores as a function of time spent disclosing emotional experiences to others, particularly when disclosure occurred several times; however, reductions were not statistically significant.

**Table 4:** Descriptive statistics of groups based on the time used for verbal emotional disclosure.

	Only a comment (*n* = 69)		More than half an hour (*n* = 42)		Several times (*n* = 122)	
	*M_1_*	*SD_1_*	*M_2_*	*SD_2_*	*M_3_*	*SD_3_*
PTSD	28.48	17.29	24.95	13.42	25.31	14.67
Intrusion	8.93	6.47	8.40	4.87	8.22	5.68
Avoidance	10.85	6.38	10.52	5.51	9.08	5.77
Anxiety	8.02	4.52	8.42	5.53	7.28	4.33
Depression	6.04	4.03	6.14	4.63	5.24	3.75

*M_*1*_, group 1 mean; SD_*1*_, group 1 standard deviation; M_*2*_, group 2 mean; SD_*2*_, group 2 standard deviation; M_*3*_, group 3 mean; SD_*3*_, GROUP 3 standard deviation; PTSD, post-traumatic stress disorder.*

## Discussion

This study was aimed at getting to know to what an extent an adolescent population is at a higher risk of experiencing traumatic situations, on the assumption that going through traumas or highly stressing situations occurs with relative frequency. Our findings indicate that a great number of adolescents have experienced traumatic situations. Results show that three out of four adolescents in the sample have undergone some sort of traumatic experience. Different studies about prevalence of exposure to trauma in adolescents found similar results (Duke et al., 2010; Alisic et al., 2014).

Additionally, the study focused on determining to what an extent the experience of traumatic events in adolescence was associated with vulnerability to certain psychological problems, such as post-traumatic stress, intrusive thoughts, avoidance behaviors, anxiety, and depression. In connection with the impact of those experiences on mental health. It was observed that those who experienced traumatic events have higher psychopathological scores (post-traumatic stress, intrusive thoughts, avoidance behaviors, anxiety, and depression) than those who did not. These findings are in agreement with the results found in other comparable research studies (Perry, 2014; Barboza Solis et al., 2015; Shi et al., 2016; Zhen et al., 2016).

Furthermore, we explored whether VED helped to reduce the psychological impact of those experiences. The major feature of the present study was that verbal disclosure can play a relevant role in reducing depressive symptoms. Because traumatic event**s** can play a role in depression vulnerability, with long-term effects (Mandelli et al., 2015), and depressive symptoms represent an important aftermath of traumatic experience (Kaltman and Bonanno, 2003), our data can be clinically relevant. In this regard, though the effect of VED on the sequels of the traumatic experience was limited, it seems to reduce depressive symptoms. As reported in other research studies about the benefits of emotional disclosure in psychological functioning and physical health, the adolescents who talked to someone about their traumatic experience showed a reduction of depressive symptoms (Peñate et al., 2010; Baikie et al., 2012; Ironson et al., 2013; Lumley et al., 2014; Blasio et al., 2015; Travagin et al., 2015; Del Pino et al., 2016).

As regards the time spent on VED results reveal a decrease in symptoms primarily when VED occurred on several occasions. The adolescents with less psychopathological symptoms were those who had talked about their traumatic experiences several times, compared to those who disclosed those experiences sporadically, or who spent little time doing so. Our results on the effectiveness of VED were mixed. There were no significant differences in symptomatology. However, improvement was observed when adolescents disclosed their traumatic experiences in an extensive way and on several occasions. These results are in line with previous studies that highlighted the modulating effects of emotional disclosure, particularly the benefits of writing repeatedly about the same event (Peñate et al., 2010; Jones, 2016).

This research study has certain limitations; we worked with a sample of volunteer adolescents without knowing about either their personal history of post-traumatic events, or the consequences that the recalled events could have for their adequate personal adjustment. It would be advisable to collect more precise information about the type, intensity, and time of the traumatic experiences, so that we can previously know the history of traumas and psychological problems, if any, and therefore have more information in order to be able to understand and explain the data.

On the other hand, in the present study there was no VED application protocol available. In this study, a formal and rigorous procedure of the VED paradigm was not carried out, but the effect of emotional expression and its intensity as a process of normalization on adverse experience was evaluated. For future research, it would be advisable to create a protocol for the procedure, characterized by its rigorous, detailed and precise application, taking into account the degree of privacy, place of sessions, instructions given to adolescents about the type of event narrated, duration and number of sessions, with the aim of implementing an experimental design with adolescents contrasting results about health and psychological well-being. Finally, as indicated by the specialized literature, the use of emotional disclosure should only be used as adjunctive therapy to other empirically supported treatments (Sloan et al., 2015).

## Conclusion

It has been demonstrated that adolescents at risk of social exclusion constitute a population vulnerable to traumatic situations, and these events may trigger serious difficulties in their psychological adjustment. Furthermore, VED by adolescents who have suffered trauma helps to reduce psychopathological symptoms, mainly depression, when adolescents repeatedly disclosed their stressful or traumatic experiences to others. In any case, findings from our descriptive data could be further explored by examining the therapeutic effects of an emotional writing disclosure procedure with a similar population in future studies.

## Ethics Statement

All children that participated had been given permission to participate by the director of their primary school, and by their parents via a signed consent form. Before data collection, children were given an oral description of the task, plus an explanation that they were free to stop the testing at any given point. The measures that were going to be used were explained. This study was carried out in accordance with the recommendations of The Ethics Committee for Research and Animal Welfare (CEIBA) of the Universidad de La Laguna, with written informed consent from all subjects. All subjects gave written informed consent in accordance with the Declaration of Helsinki. The protocol was approved by the CEIBA Committee.

## Author Contributions

WP was involved in study design. SP was involved in data collecting, analysis and manuscript drafting and revises. WP, JB, and AF were involved in data analysis, manuscript drafting and revises. We have read and approved the manuscript and agree to be accountable for all aspects of the work in ensuring that questions related to the accuracy or integrity of any part of the work are appropriately investigated and resolved.

## Conflict of Interest Statement

The authors declare that the research was conducted in the absence of any commercial or financial relationships that could be construed as a potential conflict of interest.
